# Keep meaning in conversational coordination

**DOI:** 10.3389/fpsyg.2014.01397

**Published:** 2014-12-03

**Authors:** Elena C. Cuffari

**Affiliations:** Department of Philosophy, Worcester State UniversityWorcester, MA, USA

**Keywords:** coordination, meaning making, languaging, ethics, social interaction, enaction, distributed cognition, experience

## Abstract

Coordination is a widely employed term across recent quantitative and qualitative approaches to intersubjectivity, particularly approaches that give embodiment and enaction central explanatory roles. With a focus on linguistic and bodily coordination in conversational contexts, I review the operational meaning of coordination in recent empirical research and related theorizing of embodied intersubjectivity. This discussion articulates what must be involved in treating linguistic meaning as dynamic processes of coordination. The coordination approach presents languaging as a set of dynamic self-organizing processes and actions on multiple timescales and across multiple modalities that come about and work in certain domains (those jointly constructed in social, interactive, high-order sense-making). These processes go beyond meaning at the level that is available to first-person experience. I take one crucial consequence of this to be the ubiquitously moral nature of languaging with others. Languaging coordinates experience, among other levels of behavior and event. Ethical effort is called for by the automatic autonomy-influencing forces of languaging as coordination.

It is an exciting time to be a philosopher of language, as long as one is willing to look to what is happening in the language sciences. Here one finds confirmation of the deep skepticism that loomed throughout twentieth-century reflections on language: there is no such (simple) thing. Language cannot be studied as a phenomenon that is in any way separate from sensing, acting, interacting physical bodies and complex material and social worlds. What can a growing empirical and theoretical focus on dynamic conversational behavior mean for meaning? One consequence takes the form of a philosophical question: how can we account for the inherently moral character of human interactions, even as some aspects of our interactions are well explained by self-organizing mechanisms?

In notoriously deflationary style, Richard Rorty sums up a perennial philosophical view (shared by Wittgenstein (1953) and Mead (2009), among others) when he describes language as “noises and marks,” which work by provoking other noises and marks. “To say that it [a given creature] is a language user is just to say that pairing off the marks and noises it makes with those we make will provide a useful tactic in predicting and controlling its future behavior” (Rorty, 1989, p. 15). Following Davidson, Rorty insists that language is not a medium, neither for expression nor representation (Rorty, 1989, p. 10). By seeing language as just another coping behavior with social consequences, he suggests, philosophers can get off the realism/idealism “see-saw” and thereby get to ask more practical and politically interesting questions. In particular, the upshot is that this view “… naturalizes mind and language by making all questions about the relation of either to the rest of the world *causal* questions, as opposed to the adequacy of representation and expression” (Rorty, 1989, p. 15).

Although this view is meant to espouse a “non-reductive behaviorism” (presumably with emphasis on the modifier), it can come off sounding somewhat emaciating. The “noises and marks” phrasing calls to mind Morse code, while the idea of predicting and controlling a fellow conversant evokes Terminator-type hyper-analytical visual perception that superimposes scrolling lines of data on the target object in sight. (It was the 80s, after all.) One can contrast this hollowing out of linguistic activity with a different account that was developing in the same decade—that of embodied cognitive linguistics. This research painted a radically alternative picture, that of the richly imagistic and fleshy inner life of metaphors and morphemes, all traceable to bodily structures and experiential patterns (e.g., Lakoff and Johnson, 1980; Johnson, 1987; Wierzbicka, 1988, 1996).

Interestingly, work in cognitive science today, specifically in the newly emerging paradigms of enaction, distributed cognition, and dynamical system approaches, indicates a return of the Rortyan perspective. Throughout this *social* cognitive science, the language of *coordination* increasingly is used to characterize not only social interaction dynamics and communication processes, but the workings of language itself (Clark, 1996; Fowler et al., 2008; Fusaroli et al., 2012; Dale et al., 2013, *inter alia*).

Different kinds of coordination are measured in research on language in interactional contexts. Some discuss coordination as the *alignment* of cognitive representations or conceptual schemes (Pickering and Garrod, 2004, 2014; Garrod and Pickering, 2009; Tylén et al., 2013). Conversation participants converge on representations by aligning “at many different levels, from basic motor programs to high-level aspects of meaning” (Garrod and Pickering, 2009, p. 293). Coordination understood as *physical entrainment* is also studied as potentially significant for language in its own right (Cowley, 2007; Fowler et al., 2008; Shockley et al., 2009; Riley et al., 2011). For example, Richardson et al. showed that visual attention—where people look and when—can “be coordinated on the basis of verbal contact alone” (Richardson et al., 2007, p. 407). Unintentional synchrony in seemingly non-linguistic phenomena such as posture and sway (Shockley et al., 2003), as well as speech rate (Street, 1984), vocal intensity (Natale, 1975), and pausing (Cappella and Planalp, 1981), invites analysis of linguistic interactors as constituting “joint-action systems” that can be studied as “non-decomposable units,” or “self-organized dynamical systems that emerge from the nonlinear interactions and couplings that exist between and among individuals and the environment” (Fowler et al., 2008, p. 265). Fowler et al. (2008) for example find equivalence between interpersonal and intra-personal rhythmic coordination; whether the limbs in question belong to the same person or different people, and whether they are coupled by sight or by neuro-musclar tissue, “the same dynamical entrainment processes” operate (Fowler et al., 2008). By attending to the sub-personal processes of coordination dynamics, a *supra-personal* “dialogical system” (to borrow from Steffensen, 2012) comes into view.

Recent work refines the synchrony model of coordination by introducing the idea of *synergy* (for a review, see Fusaroli et al., 2014). A synergistic notion of coordination importantly distinguishes *complementarity* rather than simultaneity as a key characteristic of successful languaging. It also emphasizes the emergent dynamics of interpersonal dyadic systems, now understood not simply as dynamically orchestrated complex machines, but as sites of social cognition. “Crucial to this synergistic model is the emphasis on dialog as an emergent, self-organizing, interpersonal system capable of functional coordination” (Fusaroli et al., 2014, p. 147).

The synergistic approach to conversational coordination dovetails well with the enactive theory of social interaction, *participatory sense-making*, which likewise puts central explanatory weight on interpersonal coordination processes and thus “allows us to claim that social interaction constitutes a proper level of analysis in itself,” one that enjoys its own autonomy or “life of its own” beyond the intentions of involved participants (De Jaegher and Di Paolo, 2007, p. 491; see also p. 494). Tracing the contours of coordination patterns and breakdowns, De Jaegher and Di Paolo describe human sociality as arising precisely in the interplay of influences between emergent interaction dynamics and the agents temporarily entrained by them (De Jaegher and Di Paolo, 2007, p. 492; see also Di Paolo and De Jaegher, 2012). Currently rounding out this coordination chorus, the distributed language approach (e.g., Thibault, 2011) pairs the early enactive autopoietic notion of languaging with the affordance paradigm of ecological psychology. “Languaging involves a complex coordination of multiple activities emphasizing the dynamics of real-time behavioral events that are co-constructed by co-acting agents” (Jensen, 2014, p. 2, this issue).

The move to complementarity, synergy, and supra-individual interaction dynamics arising from participatory coordination brings with it a slew of critical consequences for traditional analyses of conversational meaning-making, be they of philosophical or more applied linguistics stripe. The most radical implication of the coordination research is an overhaul in the definition of language itself. Language is now to be seen as a set of dynamic self-organizing processes and actions on multiple timescales and across multiple modalities that come about and work in certain domains (those jointly constructed in social, interactive, high-order sense-making). This is a very radical turn, one with many meanings. For example, on the basis of work in close kinship with these approaches, we are poised to appreciate language as multimodal (McNeill, 1992, 2005, 2012; Kendon, 2004; Streeck, 2009), and as a doing, i.e., as a “pragmatic and phonetic” rather than propositional or abstract issue (Hodges et al., 2012, p. 501). Furthermore, as Fusaroli et al. (2014) point out, taking this perspective is not merely a matter of stacking up new findings, but of clearing out old attitudes. In order to make space for proper appreciation of conversational synergy, they say we need to reject

two commonly assumed views: (1) the ultimate function [of conversational languaging] is not necessarily to reach deep mutual understanding of each other nor to converge internal representations; it is rather to realize an activity together which might or might not require deep mutual understanding (2) the function of a conversation cannot be defined on the level of the individual: the role of each individual component in a system… makes sense only within the functional organization of the dyad. (2014, p. 150)

Several key shifts are thus advocated by the synergy approach: a shift from individual to dyad in order to determine the functional teleology of a conversational interaction, and a shift from understanding the *meaning* of conversational action in terms of “deep mutual understanding” to the realization of a given and shared purpose or task.

Such shifts imply major philosophical and ethical consequences and raise a host of pressing questions (some to follow). Yet notice that these pivotal implications were more or less already there in the first-generation synchronous mechanism approach to conversational coordination. At root, the problem of coordination is “how a device of very many independent variables might be regulated without ascribing excessive responsibility to an executive subsystem” (Turvey, 1990, p. 938). Coordination means law-like patterns of movement that are emergent and self-organizing. Seeing language in this way brings about the gestalt switch that Rorty was after: language becomes a causal phenomenon, or better, a set of causal phenomena, fully on par with forces and events in the natural physical world.

The common heritage of coordination accounts of languaging interaction is the site of a significant tension, then. Precisely because language is a doing, a practical and physical as well as social and cultural activity, it finds a ready place on a continuum view of sense-making or fully embodied meaning generation, a view trained on the intrinsic normativity of always-caring, never neutral life in pursuit of life (Jonas, 1966; Di Paolo, 2005; Thompson, 2007). Recent work found in enactive, distributed, and dynamic proposals take this vantage point when they promote a nuanced and social picture of meaning-sharing. However, the paradigmatic resources of mechanism, movement, and even self-organization may be too thin, both epistemologically and ethically speaking, to account for the full significance and irreducible complexity of everyday human conversing. They only give us a birds' eye view of the story, one somehow beyond the system under observation.

I suggest that an adequately rich sense of meaning may be missed even in the synergistic coordination accounts, not because we lose track of skull-bound representations in adopting this perspective, but because we lose sight of the consequences a conversation can have for individual lives and selves (see also Kyselo, 2014, this issue). It remains to be seen if synergistic coordination gets us any further in our ability to explain how such consequences follow from the marks and noises we so perfunctorily get each other to make. (One may even notice that the coordination view of social cognition outruns the Rorty–Davidsonian dream of charitable anthropologists in the field: we are no longer predicting each other's moves, but are each and every one of us swept up in a smaller-and-larger-than-self tide of constraining and entraining languaging).

The challenge and the solution are the same: those of us interested in pursuing a radically non-representational, distributed, participatory, and behaviorally-attuned account of human languaging must work toward a better understanding of human embodied intersubjectivity as such. We are not pendulums. A conversation is more than a multimodal juggling act. But we do, in some ways, work like pendulums, and our conversations do fall into observable patterns and flows that may delight onlookers, especially those with access to multiple regression plots. It is exactly because as human social creatures we are remarkably adept at synchrony and synergy, turn-taking and rule-following, entraining each other and getting our movements hijacked installed hallway face-offs, that we must pay closer attention to what our bodies always already know how to do in conversational interactions. Empirical work supports the suspicion that just because a conversation runs like a well-oiled machine, it does not follow that interlocutors have jointly made or experienced any good sense (see Galantucci and Roberts, 2014). One possibility for paying better attention to our conversational co-enactings would be to investigate underexplored but highly relevant dimensions of our embodiment, including bodily protest, dissonance, discomfort, difference, and betrayal.

Richly intelligent and culturally elaborated as they are, our bodies can and do betray us. Frequently this betrayal comes in the form of habit. In 2007 I attended a talk that philosopher Shannon Sullivan gave on race. She spoke of one dimension of her experience of being a white southern woman: when people get verbally aggressive with her or are rude to her, she smiles. Seemingly against her will, her bodily practices carry and enact stark traces of a specific social-cultural upbringing. Despite her own frustration or discomfort, she habitually and automatically carries forward specific norms of how to be with others.

Social settings and scripts function similarly, assigning roles that play out as counterintuitive bodily actions. When I was working as a waitress in an upscale fusion restaurant about a decade ago, I once had a customer berate me and criticize my work in a way that was nonetheless perfectly polite in word choice and even in tone. But even as my body “took sides” with the insulting customer, obediently clearing his unwanted food, nodding, stepping back with a lowered head and then calmly walking away, a dissonance began to arise as a *creeping feeling*, the unsavory sense of needing to shake something off my back and shoulders, a hot tingle of anger as tears welled. There was a bodily knowledge that something in that outwardly smooth interaction had gone awry. I am not a mere billiard ball; my reactions are complex; and I do not “process” the emotional consequences of interactions immediately. With varying degrees of reflection and compassion, I can learn from experiences of bodily-emotional dissonance as I sort out the intra-individual tensions and unfold a broad range in meaning in what has transpired.

I do not know how the customer felt after this interaction on his side of things. One might imagine he felt smug and satisfied: he ultimately (and without much waiting) got what he wanted from his dining experience, and he imparted an important lesson to an ignorant girl. He sat back, comfortable, sure. He folded his hands on his belly. We both played our parts in the highly scripted ritual. We had coordinated well. But the meaning of the interaction was in no way the same for both of us.

There can be no denying the gendered and classed aspects of these examples, the distinctive contributions of personal as well as community histories. Our flesh-and-blood, inherently vulnerable, defensive embodiment senses and partially dictates the meanings that interactions have for us—consequences in terms of emotional experience, our possibilities for response and other action, our understanding. Evaluative reactions are conditioned by contexts, histories, and concern that can function as trigger points [Damasio's somatic marker hypothesis is one route toward linking social events and physiological reactions (1996)]. Non-similarity and non-identity in human embodiment thus act as content-generating resources, as each unique intelligent body-self enacts its own dance with symbols, second-order language constraints, and situational dynamics (Cuffari and Jensen, 2014). That each of us interprets events or sentences differently is a basic motivator for communication and an on-going source of meaning-granting normativity. It is through conflict, argument, and negotiation that “deep mutual understanding” gets a chance to occur (Cuffari, 2014).

From these examples we must also note and take seriously the significant *temporal* dimensions of meaning unfolding, spilling beyond the boundaries of a dyadic episode. Studies in dynamic systems may be very useful here. Language is now understood as including many timescales “from milliseconds of brain activity to hundreds of milliseconds of individual cognitive processing, seconds and minutes of interaction, months and years of language acquisition, and hundreds of years of cultural language evolution” (Rączaszek-Leonardi, 2010, p. 269). While new developments in mobile measuring technology and statistical analysis enable researchers to track the more micro of these timescales, many of these are arguably beyond the reach of what is available to phenomenological, first personal conscious experience or awareness during a languaging event. “If one agrees that language has an important function of interindividual coordination, some variables will pertain to this level, that is, the level of interaction, and may not be easily accessible to individual experience” (Rączaszek-Leonardi, 2010, p. 275). But interactive coordination, as quickly or expertly as it may take place, nonetheless has complex consequences for individual experience. Proper investigation of these unfolding consequences will likely require identifying the right timescale for this sort of meaning. Merlin Donald's “slow process” hypothesis proposes an “intermediate” time zone for “complex events that extend over several hours (for example, a game or conversation)” and points out that “adult humans typically live, plan, and imagine their lives in this time range” (Donald, 2007, p. 214). Donald sees the slow process as a uniquely human capacity that co-evolved with cultural developments precisely to “handle the cognitive demands imposed by increasingly complex distributed systems” (Donald, 2007, p. 214). The slow process hypothesis implies a “deeper background vantage point,” constituted by “a vastly extended working memory that serves as the overseer of human mental life” (Donald, 2007, p. 220). This presents a plausible physiological explanation for how individuals are simultaneously players on the great, shared stage of life while still maintaining a concrete experience and narrative sense of *my life*.

In this article I have been endorsing or performing a particular view of meaning as having to do with consequences. For example, I have used phrases like “what does this approach *mean* for language research” or “the interaction did not have the same *meaning* for both of us.” This sense cannot exhaust the rich notion of the meaning of languaging, which as we have seen, is an always on the move, dynamic phenomenon unfolding across timescales and participants. Depending on the timescale one uses in observing languaging, meaning may be apparent at the level of an event, as in the way that mothers and babies complete each other's actions (Rączaszek-Leonardi et al., 2013). In deconstructing a politician's television ad campaign, meaning may be seen at a social-systemic level. Nevertheless, the sense of meaning as “carrying forward” (Gendlin, 1962, 1997), as a series of changes or implications in phenomenologically available felt sense and action possibilities, is an important one that can and should be integrated into the social-interactive turn in cognitive science. Mark Johnson summarizes this pragmatist view of meaning, writing that human meaning is that which “concerns the character or significance of a person's interactions with their environments,”

… the meaning of a thing is in its consequences for experience—how it “cashes out” by way of experience, either actual or possible experience. Sometimes our meanings are conceptually and propositionally coded, but that is merely the more conscious selective dimension of a vast, continuous process of immanent meanings that involve structures, patterns, qualities, feelings and emotions. (Johnson, 2007, p. 10).

For some current proposals of how personal histories of culturally situated embodied experience can inform the meaning of languaging acts (wordings, gestures, improvisational performances, etc.), see Jensen, 2014; Koubová, 2014 (this issue); and Cuffari et al. (2014).

As De Jaegher and Di Paolo point out, because sense-making is “essentially embodied in action” it is “directly affected by the coordination of movements in interaction” (De Jaegher and Di Paolo, 2007, p. 497). This suggests that meaning (in the sense I mean it) can be coordinated, or more precisely, that interacting coordinates processes of meaning making (e.g., responsive embodied activities in the interaction). This observation presses the importance of ethical attunement. It tells us an important thing: language approached as coordinating also means that in conversational exchanges, emails, and elevator rides, we are constantly getting coordinating and constrained, and doing the same to others, whether or not we are aware of it. But it does not say whether coordinating another's sense-making will have good or bad outcomes, or how we are to discriminate.

Immanent, embodied dimensions of our interactions—personal experience, social position, habituated reactions, emotional and physical vulnerabilities, and temporality—are our sources of caring and evaluating. As ecological psychologist Bert Hodges tells us, “The pragmatics of languaging and language can thus largely be summarized as, learning how to be *caring* and *careful* in our speaking and listening to each other. To care and to be careful is to evaluate and select better and worse ways to move” (Hodges et al., 2012, p. 503). What will count as better or worse is sometimes immediately obvious and often an *emergent* inter-personally produced or discovered quality. But this is not always the case. The call to learning is a call to growth, improvement, and change—it does not suggest that merely going on interacting as we always do will suffice. Perhaps not every interaction “task” requires deep mutual understanding. But if it is true that “we converse in order to explore and create possibilities for doing something good together” (Hodges et al., 2012, p. 503), it seems that mutual understanding is an important element of conversational interaction. What will serve as our teacher in this crucial learning process, what can act as our normative guide, is our individual yet intersubjectively engaged embodiment.

## Conflict of interest statement

The author declares that the research was conducted in the absence of any commercial or financial relationships that could be construed as a potential conflict of interest.
